# ﻿Phylogenetic analysis of *Bettacoccina* complex (Teleostei, Osphronemidae) from Peninsular Malaysia and Sumatra Island with descriptions of two new species

**DOI:** 10.3897/zookeys.1238.142857

**Published:** 2025-05-15

**Authors:** Jiali Ding, Wen Lei, Haryono Haryono, Wentian Shi, Wanchang Zhang

**Affiliations:** 1 School of Life Sciences, Nanchang University, Nanchang 330031, China Nanchang University Nanchang China; 2 Research Center for Biosystematics and Evolution, National Research and Innovation Agency, Bogor 16911, Indonesia National Research and Innovation Agency Bogor Indonesia; 3 The Parosphromenus Project, Lage, 32792, Germany The Parosphromenus Project Lage Germany

**Keywords:** *Betta*, biodiversity, mitochondrial *cytochrome b* gene, phylogeny, taxonomy

## Abstract

The *Bettacoccina* complex is a diverse taxonomic group of fighting fish widely distributed in isolated islands in Southeast Asia. This genus is an ideal model for investigating freshwater fish evolutionary patterns and historical biogeography in the Malay Archipelago. In this study, based on principal component analysis of morphological traits, taxonomic diagnoses, and phylogenetic analysis of the mitochondrial *cytochrome b* gene, two new species are described from Sumatra Island, *Bettaiaspis***sp. nov.** and *Bettamulyadii***sp. nov.** The former has a distinctive black anal fin with a few red patches on the posterior half, while the latter is red and unspotted throughout, distinguishing them with their five or six subdorsal scales from other members of the *Bettacoccina* complex. Phylogenetic analysis of the *Bettacoccina* complex based on Cyt *b* further suggests that the speciation and present distributional pattern of this complex cannot be explained simply by the current geographic isolation of the Strait of Malacca, but by the connection-isolation scenario in the Pleistocene biogeographic context in Sundaland. This metapopulation of extant *Betta* species suggests a potential radiative evolution before the Last Glacial Maximum. These findings advance our understanding of the taxonomy and biogeographic evolution of *Betta* species in Southeast Asia.

## ﻿Introduction

The genus *Betta* (also known as fighting fish), comprises a group of small sized (< 150 mm TL) labyrinth fish distributed widely in Southeast Asia. More than 70 species have been diagnosed from this genus (Witte and Schmidt 1992; Tan and Ng 2005; Kamal et al. 2020). The *Bettacoccina* complex is a taxonomic group of small bubble-nest-building species specifically adapted to forest peat swamps with extremely low pH water conditions (Vierke 1979; Kottelat and Ng 1994a; Tan and Ng 2005; Schindler and Schmidt 2006). To date, ten species have been described in this complex from Borneo, Peninsular Malaysia, Sumatra, and affiliated islands (Vierke 1979; Schaller 1985; Schaller 1986; Witte and Kottelat 1991; Ng and Kottelat1992; Witte and Schmidt 1992; Kottelat and Ng 1994b; Tan and Tan 1994; Tan and Ng 2006; Schindler and Linke 2013). The first species described in this complex was *B.coccina* Vierke, 1979 from Jambi, Sumatra, which is now found on both sides of the Strait of Malacca in Sumatra and Peninsular Malaysia. Subsequently, *B.tussyae* Schaller, 1985, *B.persephone* Schaller, 1986, and *B.livida* Ng & Kottelat, 1992 were discovered in Peninsular Malaysia. Additionally, four species were identified from Borneo Island including *B.rutilans* Witte & Kottelat, 1991, *B.brownorum* Witte & Schmidt, 1992, *B.uberis* Tan & Ng, 2006, and *B.hendra* Schindler & Linke, 2013. Moreover, *B.miniopinna* Tan & Tan, 1994 and *B.burdigala* Kottelat & Ng, 1994 were discovered in the Bintan and Bangka islands, respectively.

Phylogenetic relationships within the genus *Betta* have been partially constructed, with species often forming distinct clades within different *Betta* complexes (Rüber et al. 2004; Kowasupat et al. 2014; Harrington et al. 2023; Yusof et al. 2024). For instance, molecular studies based on mitochondrial and nuclear DNA markers (e.g., *cytochrome b* and *RAG1*) have revealed rapid diversification events and multiple evolutionary transitions in reproductive strategies, such as mouth-brooding and bubble-nesting (Rüber et al. 2004). However, a comprehensive phylogeny encompassing all species of the *Bettacoccina* complex remains unresolved. This complex, primarily distributed across Borneo, Peninsular Malaysia, Sumatra, and adjacent islands, presents an ideal model for investigating the historical biogeography and mechanisms underlying species diversification in Southeast Asia. Resolving the phylogenetic relationships within this complex is critical for elucidating the evolutionary processes, such as Pleistocene sea-level fluctuations and habitat fragmentation, that shaped the current biodiversity patterns in this biodiversity hotspot.

**Figure 1.**
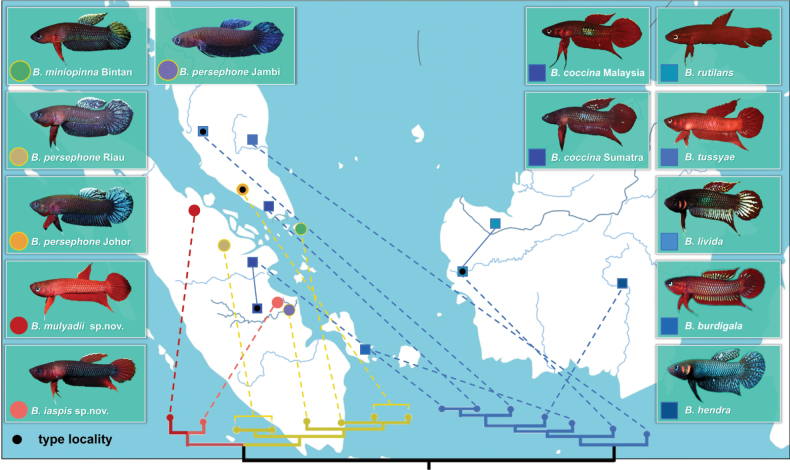
Geographic distribution of 10 species from *B.coccina* complex. The plot shows *B.iaspis* sp. nov. (pink circle) and *B.mulyadii* sp. nov. (red circle) and phylogenetic relationship among them; species from the *B.persephone* cluster are indicated by circles while species from *B.coccina* cluster are indicated by squares.

In 2012, a *Betta* species with simple red coloration was discovered in Riau, Sumatra Island (see map in Fig. 1) and was introduced into the aquarium trade under the common name “api-api”. Recently, in 2022, another *Betta* species with a blackish body and reddish fins was obtained independently by several local collectors from Jambi, Sumatra Island and circulated commercially as “*B.* sp. jade”. The phylogenetic tree based on Cyt *b* shows that these two populations from Sumatra are monophyletic groups distinct from their congeners. Genetic divergence among these specimens and other taxa also supports them as distinct species. Based on an analysis of phylogenetic separation, genetic divergence, and morphological differences, we formally describe them as two new species named *B.iaspis* sp. nov. and *B.mulyadii* sp. nov. Our study advances the understanding of the taxonomic diversity within the *Betta* genus and provides insights into the evolutionary landscape of biogeographic history in Southeast Asia.

## ﻿Materials and methods

### ﻿Sample and morphological analysis

Specimens were collected using hand nets and preserved in 95% ethanol. Holotypes were deposited at Museum Zoologicum Bogoriense (**MZB**), Research Center for Biosystematics and Evolution, National Research and Innovation Agency, Indonesia. Paratypes and non-type specimens were deposited at Museum of Biology (**NCUMB**), School of Life Sciences, Nanchang University, China. Meristic counts and morphometric measurements followed the methods of Witte and Schmidt (1992) and Shi et al. (2021). Due to challenges in measuring dehydrated and damaged specimens, we only collected data from well-preserved specimens. Measurements were performed using a stereo microscope with 0.1 mm accuracy. The meristic modal count is indicated by an asterisk (*). Species diagnosis and descriptions were guided by Witte and Schmidt (1992), Tan and Tan (1994), and Tan and Ng (2006), with species grouping based on criteria from Tan and Ng (2005). This study adopts the phylogenetic concept, which defines a species as a monophyletic group characterized by autapomorphic traits (Cracraft 1989; Warren 1992; Turner 1999; Shi et al. 2021). This concept has consistently informed the taxonomic studies of *Betta* species (Tan and Ng 2005; Tan and Ng 2006; Schindler and Linke 2012).

### ﻿Principal component analysis

To further explore the morphological differences between these two new species and existing ones, we employed principal component analysis (PCA) to examine the continuous traits across species, assessing their similarity and redundancy. Prior to analysis, all variables were standardized to a mean of zero and unit variance to ensure equal weighting. Morphological data from eight characters were analyzed via the Principal Components Analysis app in Origin 2023b with default parameters. Characters used in PCA are key morphological features employed in previous taxonomic classification including: dorsal fin rays, unpaired fin shapes, predorsal scales, subdorsal scales, lateral scales, transverse scales, body base colors and body flank color patterns. Any specimens lacking data or for which certain analyses were not relevant were excluded from the study. Morphological characteristics not directly measurable from individual specimens were coded as zero and one.

### ﻿Sequencing and phylogenetic analyses

Genomic DNA was extracted from fin tissues using the TIANamp Marine Animals DNA Kit (Tiangen, China). We amplified the Cyt *b* using primers designed by Rüber et al. (2004): DonGlu–F (5’–AACCACCGTTGTATTCAACTACAA–3’) and DonThr–R (5’–ACCTCCGATCTTCGGATTACAAGACCG–3’), yielding a product of approximately 1,140 bp. The Polymerase chain reaction (PCR) was conducted in a 25 µl reaction solution under the following conditions for 34 cycles: denaturation at 98 °C for 30 s, annealing at 60 °C for 15 s, and extension at 72 °C for 15 s. A final extension step was performed at 72 °C for 5 minutes. The PCR was performed on 57 specimens to amplify the entire Cyt *b*, followed by Sanger sequencing of the PCR products. Complete Cyt *b* sequences for each specimen were assembled from the sequenced fragments using ContigExpress (http://www.contigexpress.com).

To construct the phylogeny of the *B.coccina* complex and estimate genetic distances between the new species and their congeners, we conducted amplification and sequencing of the Cyt *b* following the method described by Rüber et al. (2004). A total of 57 specimens, representing 12 species including the two new *Betta* spp. and an outgroup taxon *Parosphromenusdeissneri* (Bleeker, 1859) were analyzed (Suppl. material 1: table S1). Phylogenetic relationships among the newly described species and other members of the *Bettacoccina* complex were inferred using Maximum Likelihood (ML) methods. In the ML analysis, the sequence alignment was 1,046 bp in length. The optimal substitution model for this sequence length was determined to be GTR using jModelTest. Phylogenetic tree was reconstructed using RAxML under the GTR model, with 1,000 bootstrap replicates. Additionally, genealogical relationships were constructed and uncorrected pairwise genetic distances (p-distance) between species were calculated using MEGA X (Kumar et al. 2018).

## ﻿Results

### ﻿The PCA based on morphometric data

We found that *Bettacoccina* complex is divided into two morphological clusters, one includes *B.coccina*, *B.livida*, *B.tussyae*, *B.rutilans*, *B.brownorum*, *B.hendra*, and *B.burdigala*, and the other consists of *B.persephone*, *B.miniopinna*, *Bettaiaspis* sp. nov. and *Bettamulyadii* sp. nov.

**Figure 2.**
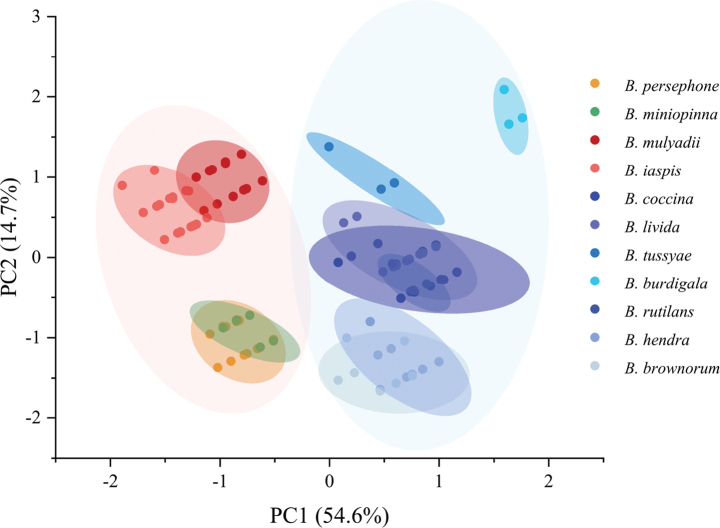
Principal component analysis based on morphological data of 11 *Betta* species. The PCA was conducted based on eight non-correlated morphological variables. The two main principal components (PC1 and PC2) explained respectively 54.6% and 14.7% of the total variation.

The first two principal components (PC1 and PC2) explained 54.6% and 14.7% of the morphological variation of the *Betta* genus, respectively (Fig. 2). The main characters between the two clusters reside in dorsal fin structure and lateral line scales. The *B.coccina* cluster has more dorsal fins rays and a longer dorsal fin than the *B.persephone* cluster. The body coloration of species in *B.persephone* cluster is mostly black, while that of the *B.coccina* cluster is mostly red. Further, the two new species from Jambi and Riau are distinguished from all other species by the analysis, confirming them as distinct species: *Bettaiaspis* sp. nov. *and Bettamulyadii* sp. nov. Besides, *B.persephone* and *B.miniopinna* are controversially indistinguishable from each other in morphospace by major principal components.

### ﻿Taxonomy

#### ﻿ Betta iaspis sp. nov.

Taxon classificationAnimaliaPerciformesOsphronemidae

AD88F610-73FC-5211-973C-50078526E61B

https://zoobank.org/9D03EDA8-554D-467C-8CBF-337337854A28

**Figure 3.**
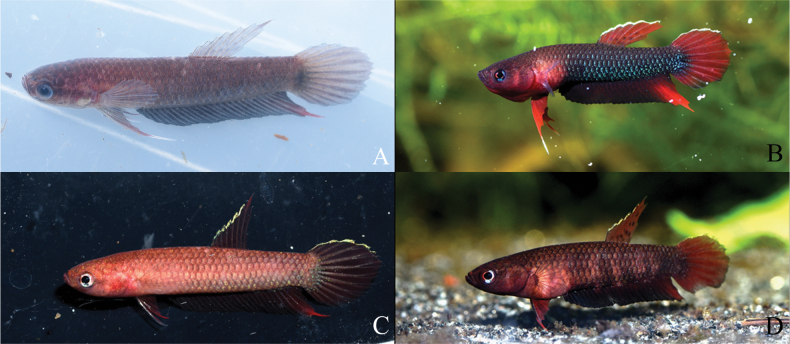
Illustrations of *Bettaiaspis* sp. nov. **A**MZB.26963, 19.5 mm SL male holotype, freshly preserved after collection in field (laterally inverted) **B** male, live coloration, not preserved (laterally inverted) **C** male holotype, in stress coloration immediately after capture (laterally inverted) **D** female, in courtship coloration, not preserved (laterally inverted).

**Figure 4.**
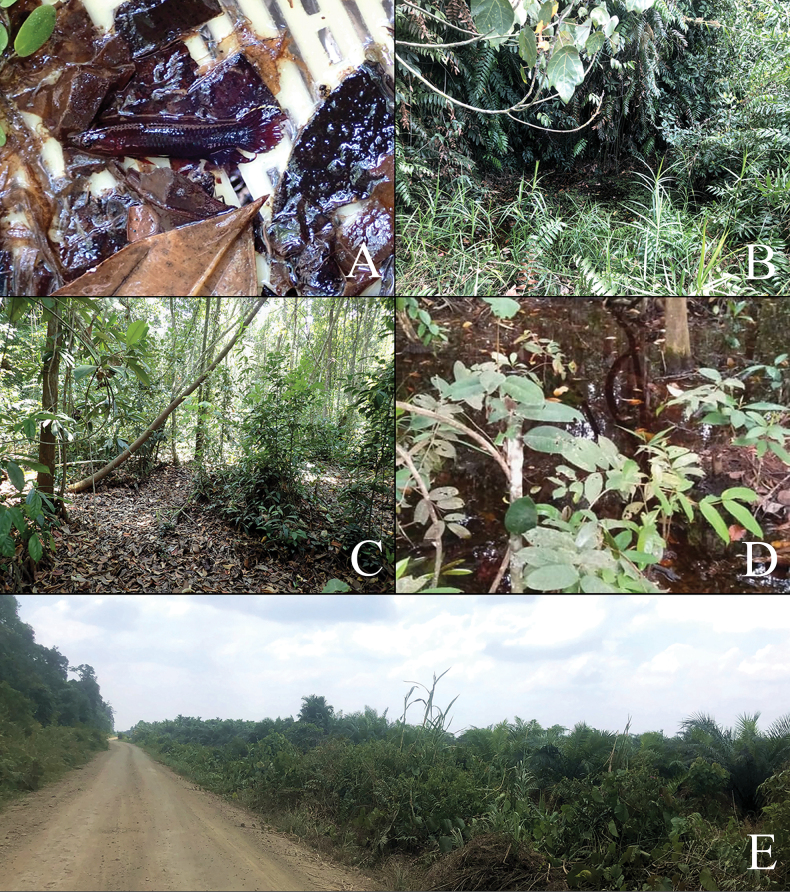
Type locality photographs for *B.iaspis***A** freshly collected specimens of male from type locality in dry season (laterally inverted), coll. Mulyadi Tjoa Hong Tjai **B** photograph of remaining puddles in the forest in dry season, photograph by Mulyadi Tjoa Hong Tjai 2023 **C** the surface of the type locality appear to be dried-up in dry season, while the mud beneath the thick layer of dead leaves often remain moist **D** this area will turn to swamp in rain season, photograph by Agus 2022 **E** type locality in Jambi, a small forest swamp directly adjacent to a huge oil-palm plantation.

Figs 3 , 4 , Suppl. material 2: fig. S1

##### Type material.

***Holotype*.**MZB.26963, 22.0 mm SL, male; Indonesia, Sumatra Island, Jambi, forest peat swamp; colls. Mulyadi Tjoa Hong Tjai, Nov. 2023. The exact locality is withheld to avoid potential pressure on the wild population of ornamental fish industry. Qualified researchers can request the information from the first author or MZB. ***Paratypes*.**NCUMB.65334, 30 specimens, 17.7–23.9 mm SL; same data as for holotype; colls. Mulyadi Tjoa Hong Tjai Jun. 2022.

##### Diagnosis.

*Bettaiaspis* sp. nov. differs from its congeners in the *B.coccina* group by the following unique combination of characters: less dorsal-fin rays (8–10*) and subdorsal scales (5–6*); shorter dorsal–fin base (7.5–19.1% SL, mean 11.7%); male with dark blackish body; without green iridescent mid-lateral body patch; dorsal-, pelvic- and caudal fins red without significant marks; blackish anal fin with reddish patches on posterior part.

##### Description.

Morphometric and meristic data are summarized in Table 1. General appearances presented on Fig. 3. Head rounded and small. Body slender (at dorsal-fin origin 18.2%–22.3% SL, mean 19.9%) not compressed at caudal peduncle (11.9%–20.5% SL). Dorsal fin narrow (total 8–10* rays), base short (7.5–19.1% SL with 5–6* subdorsal scales) and placed significantly far back (predorsal length 57.8–67.7% SL). Dorsal fin pointed with elongated posterior rays, sometimes reaching caudal-fin base in mature males. Anal fin situated ~ ½ body (preanal length 39.7%–47.0% SL), base long (47.9%–57.8% SL). Anal fin with total 27*–28 rays, pointed, posterior rays elongated, often reach half-length of caudal fin in mature males. Caudal fin lanceolate in males, rounded in females, with i-ii rudimentary, I simple principal, 4+5 branched principal, I simple principal, i-ii rudimentary rays (modal ii-I-4+5-I-ii). Pectoral fin rounded with 12–14 (modal 13) rays. Pelvic fin with one spine, one simple and four branched rays, simple ray filamentous. Lateral scales 28*–30, plus two or three scales on caudal-fin base; predorsal scales 19–21*; postdorsal scales 9–11 (modal 10); 7–8* scales in transverse series at dorsal fin origin.

**Table 1.** Morphometric and meristic data of *Bettaiaspis* sp. nov. (*n* = 31, MZB.26963, NCUMB.65334) from Jambi, Sumatra, and *Bettamulyadii* sp. nov. (*n* = 29, MZB.26964, NCUMB.65326) from Riau, Sumatra, Indonesia.

Morphometrics	*Bettaiaspis* sp. nov.		*Bettamulyadii* sp. nov.	
	Holotype	Paratype	Holotype	Paratype
		minimum–maximum (mean ± standard deviation SD)		minimum–maximum (mean ± standard deviation SD)
Standard length (mm)	22.0	17.7–23.9 (21.7 ± 1.6)	18.5	22.4–28.4 (24.9 ± 1.8)
As % Standard length				
Total length	132.3	112.0–131.3 (124.9 ± 4.1)	135.1	119.1–129.6 (124.3 ± 2.6)
Predorsal length	67.7	57.8–66.1 (61.6 ± 2.0)	64.9	58.1–63.5 (60.8 ± 1.4)
Postdorsal length	22.7	21.8–32.2 (25.3 ± 2.6)	24.3	21.3–28.9 (24.1 ± 2.2)
Preanal length	45.5	39.7–47.0 (42.6 ± 1.7)	42.7	38.1–43.5 (40.2 ± 1.6)
Head length	28.6	21.6–28.8 (25.1 ± 1.6)	26.9	21.2–26.2 (23.7 ± 1.2)
Body depth at dorsal-fin origin	20.0	18.2–22.3 (19.8 ± 1.1)	16.9	18.7–21.7 (23.7 ± 1.2)
Pelvic-fin length	37.7	18.3–32.8 (24.8 ± 4.3)	31.4	19.6–36.5 (28.0 ± 4.6)
Anal-fin base length	50.9	47.9–57.8 (52.7 ± 2.0)	48.1	50.7–58.9 (54.2 ± 2.0)
Dorsal-fin base length	12.8	9.87–15.1 (11.9 ± 1.3)	13.0	9.9–15.1 (12.7 ± 1.3)
Caudal peduncle depth	12.4	11.9–15.6 (14.1 ± 0.8)	12.5	12.9–15.2 (14.1 ± 0.7)
In % Head length (mean ± SD)				
Orbit diameter	27.1	25.5–34.8 (29.0 ± 2.6)	30.1	24.6–37.5 (27.9 ± 3.0)
Postorbital length	50.8	49.1–62.0 (55.2 ± 3.0)	47.8	50.9–57.8 (54.9 ± 1.9)
Interorbital distance	42.8	31.6–45.3 (39.2 ± 3.6)	41.1	34.4–46.4 (40.8 ± 3.5)
Snout length	17.5	12.0–19.0 (15.8 ± 1.7)	15.6	14.1–19.3 (16.7 ± 1.4)
MERISTICS (Total counts)		minimum–maximum		minimum–maximum
Anal-fin rays	27	27–28	27	27–29
Dorsal-fin rays	10	8–10	8	8–10
Caudal-fin rays	i-I-4+5-I-i	i-I-4+5-I-i	i-I-4+5-I-i	i-I-4+5-I-i
Pelvic-fin rays	6	6	6	6
Pectoral-fin rays	12	12–14	13	12–14
Subdorsal scales	6	5–6	5	5–6
Transverse scales	8	8	8	8
Lateral scales	30	28–30	30	29–30
Predorsal scales	21	19–21	21	19–21
Postdorsal scales	10	9–11	10	9–12

##### Live coloration.

***Male*** (Fig. 4). Head reddish brown, dorsum dark brown. Opercle without distinct bar in neutral mood, two barely recognizable pale reddish twin bars on opercle in aggressive or breeding mood. Iris with iridescent bluish patches. Anterior edge of body reddish brown, belly area brown, posterior edge of body after anus blackish with pale flush of iridescent blue (less distinct reddish brown in stressed or preserved specimens, Fig. 4C), dorsum dark brown. No distinct dark stripes or iridescent patches on body flank. Dorsal fin simple red with a bright bluish margin (blackish basal spots presented on inter-radial membrane of some specimens). Caudal fin simple red with a bright bluish margin on the upper half. Anal fin blackish with reddish patches on posterior part. Pectoral fin hyaline. Pelvic fin red with whitish filamentous ray.

***Female*** (Fig. 4D). Head and body coloration similar to male, but blackish part less intense. All fins coloration similar to male, but blackish black spots more intense on dorsal fin. In breeding condition, dark stripes can be observed on the body.

##### Preserved coloration.

***Male*** (Fig. 3, Suppl. material 2: fig. S1): Head dark brown, dorsum blackish, no bar present on opercle. Anterior edge of body yellowish brown, posterior edge of body after anus pale blackish (dark brown in juvenile specimens), dorsum dark brown. Dorsal fin simple red without distinct margin (indistinct blackish spots presented on inter-radial membrane of some specimens). Caudal fin simply red without distinct margin. Anal fin blackish with reddish patches on posterior part. Pectoral fin hyaline. Pelvic fin red with hyaline filamentous ray.

***Female*** (Suppl. material 2: fig. S1): Head and body coloration similar to male, but posterior edge of body after anus dark brown. All fins coloration similar to male.

##### Comparison.

*Bettaiaspis* sp. nov. can be easily distinguished from other members of the *B.coccina* group by the following combination of characteristics: shorter dorsal-fin base (7.5–19.1% SL), fewer subdorsal scales (5–6) and its unique caudal-fin coloration (blackish with reddish patches on posterior part); it also differs from its most similar congeners *B.persephone* and *B.miniopinna* by the presence of red dorsal- and caudal fins (vs dark greenish/bluish); filamentous elongated posterior rays of anal fin (vs pointed but not filamentous). It can also be distinguished from *B.burdigala*, *B.coccina*, *B.livida*, *B.tussyae*, and *B.uberis* by lack of iridescent greenish/bluish streaks/patches on inter-radial membrane of unpaired fins (vs presence); differs further from *B.burdigala* and *B.uberis* by fewer dorsal-fin rays (8–10* vs 14–17), from *B.coccina* by whitish pelvic fin tips (vs black), from *B.livida* by fewer lateral scales (28*–30 vs 30–31); from *B.tussyae* by presence of iridescent blue on body flank (vs absence). It also differs from *B.brownorum* and *B.rutilans* by blackish body color (vs reddish magenta), further distinct from *B.brownorum* by lack of iridescent mid-lateral blotch (vs presence), from *B.rutilans* by presence of bright bluish margin on dorsal- and caudal fin (vs absence/indistinct). It can be differentiated from *B.hendra* by absence of distinct bar on opercle (vs prominent red parallel bars), less transverse scales 7–8* (vs 8–10), blackish or reddish unpaired fins (vs greenish/bluish).

##### Distribution.

*Bettaiaspis* sp. nov. is currently only known from one single forest peat swamp in Jambi, Sumatra Island (Fig. 1), which is adjacent to a huge oil palm plantation.

##### Etymology.

A Latin noun *iaspis* is derived from the Greek *ἴασπις*, for the gemstone jasper, which is usually red or green/blue in color, referring to the distinct combination of the fish’s iridescent bluish/greenish body and reddish fins.

##### Field notes.

Specimens were collected from a small forest peat swamp, which can be partly dried-up in the dry season. Similar to its congeners *B.persephone* in Malaysia (Schaller 1986), *Bettaiaspis* sp. nov. also survives through the dry season, particularly in the dried-up area of the swamp, by hiding in the moist mud beneath the dead leaves on the ground (Fig. 3). In the rainy season, when the habitat is filled again with water, they come back to surface and reproduce normally like other members of the group. Certain water parameters were recorded in dry season: pH 5.0, total dissolved solids in water 12 ppm, water temperature 29.8 °C.

All syntopic fish species recorded from the type locality are as follows: *Bettasimorum* Tan & Ng, 1996, *Borarasmaculatus* (Duncker, 1904), *Rasboraeinthovenii* (Bleeker, 1851), *Sphaerichthysosphromenoides* Canestrini, 1860.

##### Conservation status.

Oil-palm plantations and coal mining activities have severely affected the natural habitats in Jambi, especially forest peat swamps along Batang Hari River (Rustiadi et al. 2018). The Lagan region, the distribution area of *Bettaiaspis* sp. nov., is facing the same challenges. During the field survey in 2023, we noticed that the type locality is immediately adjacent to a huge oil-palm plantation (Fig. 4C), whose irrigation activities have aggravated the drought of the forest swamp. Although the species has evolved a successful survival strategy against nature drought, it will fail eventually if the continuing deterioration of the habitat’s hydrological conditions cannot be stopped by proper conservation efforts. Thus, following the IUCN Red List Categories and Criteria (ver. 3.1), we propose *Bettaiaspis* sp. nov. be listed as Critically Endangered B2ab (iii), based on its very restricted distribution area in a single forest peat swamp (< 30 km^2^) and the fact that this habitat has already been facing direct threats from human activities.

##### Molecular analysis.

The consensus phylogenetic tree based on the Cyt *b* suggests that *Bettaiaspis* sp. nov. is a monophyletic group distinct from its sister group *B.mulyadii* sp. nov. by an uncorrected p-distance of 4.35% (Fig. 7; Suppl. material 1: table S2). *Bettaiaspis* sp. nov. is also distinct from other congeners in the same branch for which Cyt *b* is available, with a p-distance ranging from 5.40%–6.32%, and significantly distinguished from the remaining species in the other branch with a p-distance ranging from 19.2%–22.0%. These results indicate that the genetic differences between the new species and its congeners exceed the intraspecific differences observed (< 2% in the current study, < 1% in related labyrinth fish species like *Parosphromenus* spp. [Shi et al. 2021] or K2P < 0.5% in *Channaargus* [Zhou et al. 2019]) (< 2–3% in previously published comparable data by COI gene by Fahmi et al. 2020 and Panijpan et al. 2014). Morphologically, *Bettaiaspis* sp. nov. differs from all known *Betta* species (see above Diagnosis and Comparison). Thus, based on both a significant morphological diagnosis and a Cyt *b* divergence consistent with that, we are confident that these specimens from Jambi, Sumatra, represent a valid novel species.

#### ﻿ Betta mulyadii sp. nov.

Taxon classificationAnimaliaPerciformesOsphronemidae

CBF1B16A-91C8-5A5F-AA89-D5FF94D7F18A

https://zoobank.org/C327437A-2C23-4255-BF77-EE2310DCD5A8

**Figure 5.**
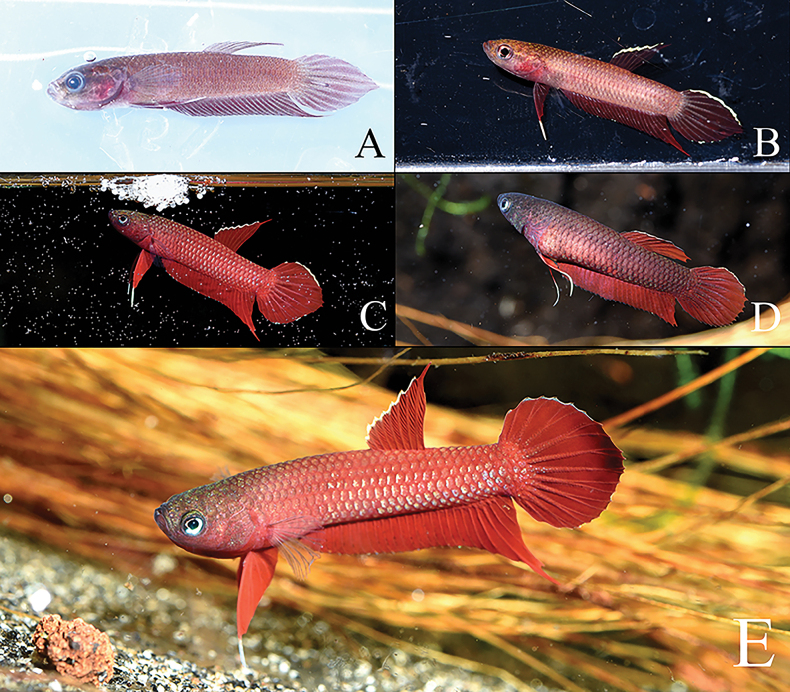
Illustrations of *Bettamulyadii* sp. nov. **A**MZB.26964, 18.5 mm SL male holotype, freshly preserved **B** male holotype, in stress coloration immediately after capture **C** breeding male with a bubble-nest, not preserved (laterally inverted) **D** female, in courtship coloration, not preserved **E***B*male, live coloration, not preserved (laterally inverted).

**Figure 6.**
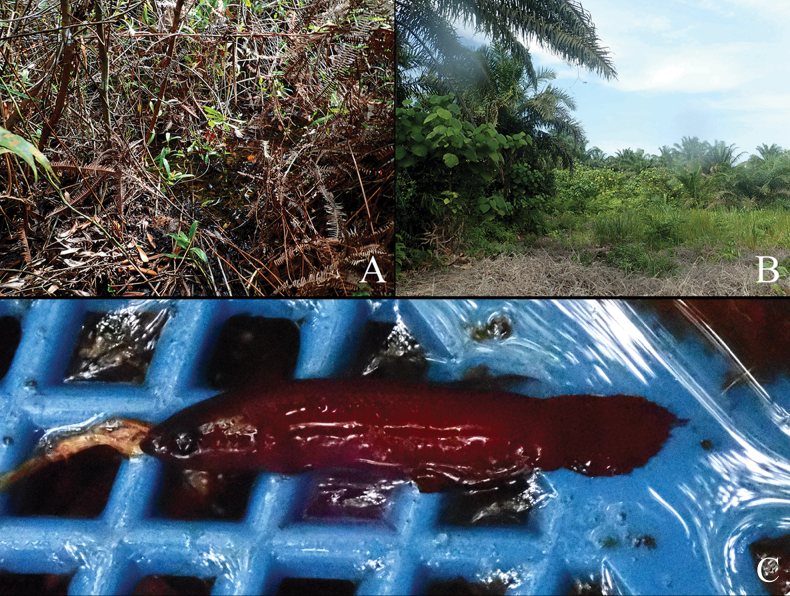
Type locality photographs for *B.mulyadii***A** swamp shaded by secondary shrubs **B** a tiny swamp surrounded by a huge oil-palm plantation **C** freshly collected specimens of male *B.mulyadii* from type locality, coll. Mulyadi Tjoa Hong Tjai.

Figs 5 , 6 , Suppl. material 2: fig. S2

Betta sp. Riau Red—Linke 2014: 228.

##### Type material.

***Holotype*.**MZB.26964, 18.5 mm SL, male; Indonesia, Sumatra Island, Riau, Oil palm plantation; colls. Mulyadi Tjoa Hong Tjai, Nov. 2023. The exact locality withheld to avoid potential pressure on the wild population of ornamental fish industry. Qualified researchers can request information from the first author or MZB. ***Paratypes*.**NCUMB.65326, 28 specimens, 22.4–28.4 mm SL; same data as for holotype; colls. Mulyadi Tjoa Hong Tjai & Johan Raharjo. Jun. 2022.

##### Diagnosis.

*Bettamulyadii* sp. nov. differs from its congeners in the *B.coccina* group by the following unique combination of characters: fewer dorsal-fin rays (8*–10) and subdorsal scales (5*-6); shorter dorsal-fin base (9.89–15.1% SL, mean 12.5%); male with reddish body; without green iridescent mid-lateral body patch; unpaired fins red without significant marks (dorsal and caudal fins with bright bluish margins).

##### Description.

Morphometric and meristic data are summarized in Table 1. General appearances presented in Fig. 5. Head rounded and small. Body slender (at dorsal-fin origin 18.7%–22.0% SL, mean 19.9%), not compressed at caudal peduncle (12.9%–16.5% SL). Dorsal fin narrow (total 8*–10 rays), base short (9.89–15.1% SL with 5*–6 subdorsal scales) and placed significantly far back (predorsal length 58.1–65.9% SL). Dorsal fin pointed with elongated posterior rays, sometimes reaching caudal-fin base in mature males. Anal fin situated ~ ½ body (preanal length 38.1%–43.5% SL), base long (48.9%–58.9% SL). Anal fin with total 27*–29 rays, pointed, posterior rays elongated, sometimes reach half-length of caudal fin in mature males. Caudal fin lanceolate in males, rounded in females, with i-ii rudimentary, I simple principal, 4+5 branched principal, I simple principal, i-ii rudimentary rays (modal i-I-4+5-I-i). Pectoral fin rounded with (12–14, modal 13) rays. Pelvic fin with one spine, one simple and four branched rays, simple ray filamentous. Lateral scales 29–30*, plus two or three scales on caudal-fin base; predorsal scales 19–21*; postdorsal scales 9–12 (modal 10); 7–8* scales in transverse series at dorsal fin origin.

##### Live coloration.

***Male*** (Fig. 5B, C, E). Head reddish with iridescent greenish/golden patches, dorsum reddish with iridescent greenish/golden patches. Opercle without distinct bar in neutral mood, two barely recognizable pale reddish twin bars on opercle in aggressive or breeding mood. Iris with iridescent bluish patches. Body uniformly reddish with weak iridescent blue patches on some scales (absent in stressed or preserved specimens), dorsum reddish with iridescent greenish/golden patches. No distinct dark stripes or iridescent patches on body flank. Dorsal-fin simply red with a bright bluish margin. Caudal fin simple red with a bright bluish margin on the upper half (sometimes also present on the lower half). Anal fin simply red (rarely with a bluish margin). Pectoral fin hyaline. Pelvic fin red with whitish filamentous ray.

***Female*** (Fig. 5D). Head and body coloration similar to male, but less reddish. All fin colorations similar to male, but bluish margin less distinct.

##### Preserved coloration.

***Male*** (Suppl. material 2: fig. S2): Head yellowish brown, dorsum brownish, no bar present on opercle. Body and dorsum uniformly yellowish brown. Unpaired fin uniformly dark reddish without distinct margin. Pectoral fin hyaline. Pelvic fin red with hyaline filamentous ray.

***Female*** (Suppl. material 2: fig. S2): Head and body coloration similar to male. All fin colorations similar to male, but more brownish.

##### Comparison.

*Bettamulyadii* sp. nov. can be easily distinguished from the closely related phylogenetic sister species *Bettaiaspis* sp. nov. by the following combination of characters: uniformly reddish body (vs blackish), simple reddish anal fin (vs blackish with reddish patches on posterior part), dorsal-fin inter-radial membrane without dark marks (vs present). It is also distinct from other congeners of the same phylogenetic branch *B.persephone* and *B.miniopinna* by the presence of red dorsal- and caudal fins (vs dark greenish/bluish); filamentous elongated posterior rays of anal fin (vs pointed but not filamentous). It differs from its most morphologically similar species *B.rutilans* by the presence of a bright bluish margin on dorsal and anal fins (vs absence/indistinct), elongated anal-fin posterior rays reaching half-length of caudal fin in mature males (vs slightly pointed but not significantly elongated), fewer dorsal-fin rays (8*–10 vs 11–12*), fewer subdorsal scales (5*–6 vs 9*–10) and shorter dorsal-fin base (7.5–19.1% SL vs 20–23%). It also differs from *B.burdigala*, *B.coccina*, *B.livida*, *B.tussyae*, and *B.uberis* by lack of iridescent greenish/bluish streaks/patches on inter-radial membrane of unpaired fins (vs presence); differs further from *B.burdigala* and *B.uberis* by fewer dorsal-fin rays (8*–10 vs 14–17), from *B.coccina* by whitish pelvic-fin tips (vs black), from *B.livida* and *B.tussyae* by fewer lateral scales (29*–30 vs 30–31). It can be differentiated from *B.brownorum* by the lack of an iridescent mid-lateral blotch (vs presence); from *B.hendra* by a reddish body and fins (vs iridescent bluish/greenish), absence of distinct bar on opercle (vs prominent red parallel bars), fewer transverse scales 7–8* (vs 8–10).

##### Distribution.

*Bettamulyadii* sp. nov. is currently known only from an area in Riau, Sumatra Island (Fig. 1).

##### Etymology.

The species is named after Mulyadi Tjoa Hong Tjai, who discovered this species and contributed much first-hand field information on this genus during the last 30 years. Previously the species was widely known by the common name “api-api” given by the discoverer, which means flame/fire in Bahasa Indonesia referring to the reddish body and lanceolate caudal fin.

##### Field notes.

The species was first recorded by Mulyadi Tjoa Hong Tjai in Duri in 2012. Currently it is still only found in this area, which is severely disturbed by human activities. Most of the nearby regions have been converted into oil-palm plantations or occupied by oil wells. Thus, we have not yet been able to record this species outside the type locality, which is a broken tiny swamp located inside an oil-palm plantation and adjacent to several running oil wells (Fig. 6). Further studies will be necessary to explore potential remaining population and habitats of this endangered species.

All the syntopic fish species recorded from the habitats include Bettacf.pugnax (Tan & Ng, 2005) and *Rasboraeinthovenii*.

##### Conservation status.

Oil-palm plantations and the petroleum industry have severely destroyed the environment in Duri, especially forest peat swamps. During the field survey in 2023, little original forest peat swamps could be found in this region. The type locality of the new species is in poor condition, original forest no longer exists, and the remaining swamp is shaded by secondary shrubs. Biodiversity is very low, which is expected since it is inside a large oil-palm plantation (Fig. 6B). Few conservation efforts for freshwater fish are known there, since it is also a petroleum industry area. Immediate in-situ and ex-situ conservation is highly recommended for this species. Thus, following the IUCN Red List Categories and Criteria (v. 3.1), we propose *Bettamulyadii* sp. nov. be listed as Critically Endangered B2ab (iii), based on its very restricted distribution area in a single swamp (< 5 km^2^) and the extremely high likelihood of becoming extinct due to the current existence of surrounding oil-palm plantations and oil wells.

##### Molecular analysis.

**Figure 7.**
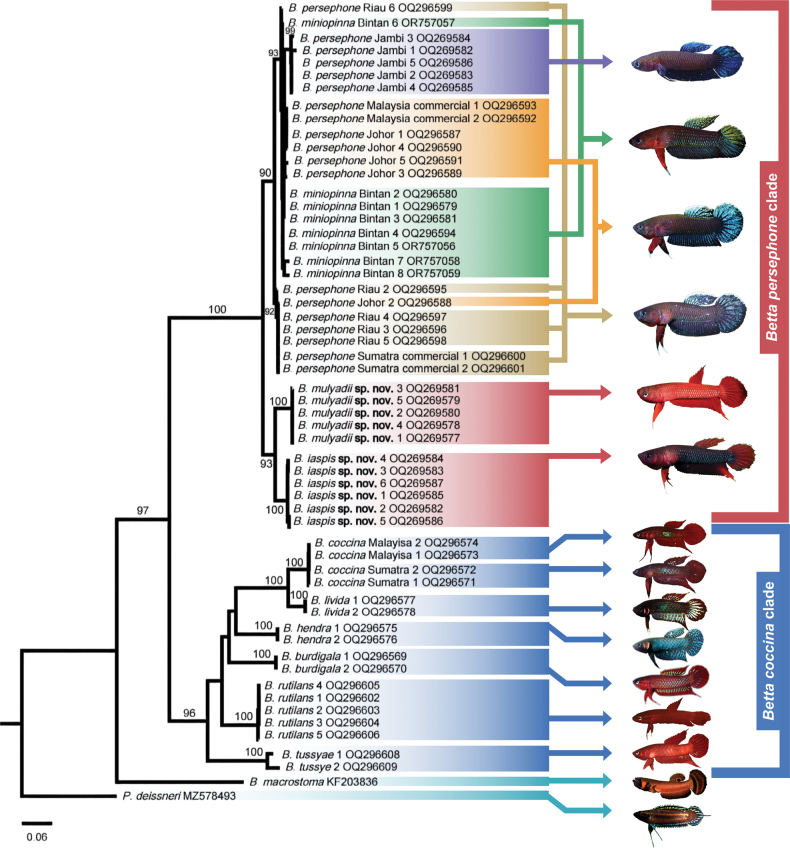
Phylogenetic tree of *Betta* species based on Cyt *b* using ML approaches. The two new species in the phylogenetic tree are highlighted in red (photograph of *Bettapersephone* Jambi: Mulyadi Tjoa Hong Tjai).

The consensus phylogenetic tree based on the mitochondrial Cyt *b* suggests that *Bettamulyadii* sp. nov. is a monophyletic group distinct from its sister group *Bettaiaspis* sp. nov. by an uncorrected p-distance of 4.35% (Fig. 7; Suppl. material 1: table S2). *Bettamulyadii* sp. nov. is also distinct from other congeners in the same branch for which Cyt *b* is available with a p-distance ranging from 5.75% to 7.19% (Suppl. material 1: table S2), and distinguished from the remaining species in the other branch with a p-distance ranging from 19.2%–22.0% (Suppl. material 1: table S2). These results suggest that genetic differences among the new species and its congeners are indicative of divergence at a species level. *Bettamulyadii* sp. nov. also differs from all known *Betta* species in its unique morphological characters (see above Diagnosis and Comparison). Thus, based on both molecular and morphological analysis in the current study, this fish from Riau, Sumatra is formally recognized as a distinct species.

### ﻿Phylogenetic analyses

The ML tree reconstructed based on Cyt *b* sequences revealed a highly resolved topology for the *Bettacoccina* complex, with all key branches supported by bootstrap values > 90%, providing a robust phylogenetic relationship (Fig. 7). We found that the 11 *Betta* species within this complex formed two major clades (the *B.coccina* and *B.persephone* clades) in the phylogenetic tree, with high consistency to the PCA results (Fig. 2).

Two sub-clades were observed in the *B.persephone* clade (Fig. 7). *Bettaminiopinna* from Bintan island and *B.persephone* from Sumatra and peninsular Malaysia form a single sub-clade in the phylogenetic tree. However, we found that *B.miniopinna* did not form a monophyletic group, but a polyphyletic group with respect to *B.persephone*. Meanwhile, similar cases occur in *B.persephone* from Riau and peninsular Malaysia, which also did not form any monophyletic group but intersected with each other and with *B.miniopinna*. The two new species, *Bettaiaspis* sp. nov. and *Bettamulyadii* sp. nov., independently form a monophyletic branch. *Bettaiaspis* sp. nov. and *B.mulyadii* sp. nov. are evolutionarily closely related to *B.persephone*, which is consistent with the PCA results and taxonomic diagnosis. Therefore, the phylogenetic analyses indicated that *B.iaspis* sp. nov. and *B.mulyadii* sp. nov. are independent species belonging to the *Bettacoccina* complex.

In the *B.coccina* clade, the phylogenetic analyses indicates that the samples of *B.coccina* from opposite sides of the Malacca Strait, southern part of peninsular Malaysia, and the northern side of Sumatra, fall in the same branch with low genetic distances (Figs 1, 7). *Bettatussyae* from the eastern side of peninsular Malaysia is morphologically close to the two sister species *B.coccina* and *B.livida*, both of which inhabit Sumatra and the western sides of peninsular Malaysia. However, *B.tussyae* did not fall into the same branch with *B.coccina* and *B.livida* in the phylogenetic tree (Fig. 7). The same phenomenon was also observed between *B.rutilans* and *B.hendra*, which are distributed on western and southern Borneo, respectively, but are phylogenetically apart from each other. This indicates that the *B.coccina* clade presents a potential biogeographical radiation of the *Betta* genus on the Malay Archipelago.

## ﻿Discussion

### ﻿*Betta speciation* older than Last Glacial Maximum

In this study, we noticed that *B.persephone* and *B.coccina* are distributed on both sides of the Strait of Malacca, in Sumatra and peninsular Malaysia (Fig. 1). The genetic analysis revealed that the average intraspecies genetic distance between *B.persephone* populations from Sumatra and Malaysia is just 1.67% (Suppl. material 1: table S2), despite their separation by Strait of Malacca (Fig. 1; Suppl. material 1: table S2). Conversely, *Bettaiaspis* sp. nov. and *Bettamulyadii* sp. nov. exhibit a genetic distance of 4.35% (Suppl. material 1: table S2), significantly higher than the former, despite their co-location on the same island. Similarly, minor intraspecies differentiation was noted between *B.coccina* from Malaysia and Sumatra, with a genetic distance of only 1.92%. According to Mueller (2006), a clade with an inter-clade and intra-clade genetic distance ratio exceeding 10% might be classified as a new clade, while intra-species differences greater than 3% could suggest speciation or the emergence of new species. Therefore, despite strict geographic isolation, the populations of *B.persephone* and *B.coccina* from the Peninsular Malaysia and Sumatra are not distinct species, but isolated populations in the same monophyletic branch with less genetic distances of the same species. This cross-sea distribution suggests that these species diverged before the geographic isolation caused by the Strait of Malacca, which formed after the Last Glacial Maximum (LGM) (Lohman et al. 2011).

During the early Pleistocene epoch (~ 2.58 million years ago), the islands of Sumatra, Java, and Borneo and peninsular Malaysia coalesced from previously isolated landmasses due to sea level regression, forming the vast unified landmass known as Sundaland (Lohman et al. 2011). This period of low sea levels persisted until the LGM (~ 21,000 years ago), during which the Sunda Shelf was extensively exposed, creating a land bridge across the Strait of Malacca without significant geographic barriers (Hanebuth et al. 2011). If *B.persephone* and *B.coccina* had already formed and existed widely in the Sunda Shelf at this period, it explains why different populations in the same monophyletic branch with low genetic distances of the same species are now distributed on both sides of the sea. Harrington et al. (2023) also demonstrated that the diversification of *Betta* species was initiated prior to the singularization of Sundaland in Southeast Asia, aligning with our findings of pre-LGM divergence in the *B.coccina* complex.

Approximately 12,000 years ago, with the onset of the Holocene, a warming climate led to the melting of glaciers, causing sea levels to rise and initiating geographic isolation in the Malay Archipelago, such as between peninsular Malaysia and Sumatra, eventually forming the modern Strait of Malacca (Bird et al. 2007; Culver et al. 2015), which leads to the current demographic status of *B.coccina* complex. This separation of different populations of the same species, *B.persephone* and *B.coccina*, is thus, a simple de facto geographic isolation rather than a genuine biogeographic reason for the speciation of these species.

Traditional theories of allopatric speciation suggest that significant geographic isolation is required for speciation to occur (Mayr 1942; Coyne and Orr 2004; Shafer and Wolf 2013). Such conventional doctrines have predominantly posited that geographic isolation leading to reproductive isolation constitutes a pivotal mechanism underpinning speciation and diversification. However, this alone is not the simple answer to the speciation of *B.coccina* complex, which is not due to the most recent geographic isolation (allopatric speciation), but rather stems from a potential radiative evolution on the ancient Sunda continent. An illustrative example is provided by the adaptive radiation of the Darwin finches on the Galapagos archipelago, which evolved to occupy divergent ecological niches within a relatively abbreviated temporal frame (Lamichhaney et al. 2015). The historic biogeographical context of the Malay Archipelago and our phylogenetic analyses into the *B.coccina* complex revealed that this group of labyrinth fish constitutes a well-distributed metapopulation in the ancient Sundaland, which further indicates its current demographic status is not a simple result of the contemporary geographic isolation but a radiative evolution before LGM.

### ﻿*Bettaminiopinna* is probably not a valid phylogenetic species

Tan and Tan (1994) identified *B.miniopinna* as a new species on Bintan Island based on morphological analysis and noted its high similarity to *B.persephone* in morphological features and behavioral patterns. However, PCA results indicated that these two species fall within the same morphological cluster and are quite indistinguishable (Fig. 2), suggesting a lack of distinct autapomorphic traits between them. The Cyt *b*-based phylogenetic tree revealed that various topotype samples of *B.miniopinna* did not form a monophyletic group, instead mixing with *B.persephone* samples from various locations in Sumatra and Peninsula Malaysia to form a complicated polyphyletic group (Fig. 7). Instead, *B.miniopinna* and *B.persephone* together constitute a clear monophyletic clade. Furthermore, genetic distance analysis showed that the maximum genetic distance between Bintan Island samples (topotypes of *B.miniopinna*) and *B.persephone* samples is 1.94% (average 1.42%), whereas it is even higher, at 2.52% (average 1.67%), among *B.persephone* populations across the Malacca Strait from Sumatra and Peninsula Malaysia (Suppl. material 1: table S2). According to the definition of phylogenetic species and our previous studies (Hebert et al. 2003; Shi et al. 2021), the interspecific distance between two valid phylogenetic species should exceed their intraspecific distances. Thus, our studies suggest that *B.miniopinna*, lacking distinct autapomorphic traits and forming a non-monophyletic group with *B.persephone*, does not qualify as a separate phylogenetic species but is instead a population of *B.persephone* from Bintan Island.

### ﻿Comparative material

For details of examined comparative materials see Suppl. material 1.

### ﻿Nomenclatural acts registration

The electronic version of this article in portable document format represents a published work according to the International Commission on Zoological Nomenclature (ICZN), and hence the new names contained in the electronic version are effectively published under that Code from the electronic edition alone (see Articles 8.5–8.6 of the Code). This published work and the nomenclatural acts in contains have been registered in ZooBank, the online registration system for the ICZN. The ZooBank LSIDs (Life Science Identifiers) can be resolved and the associated information can be viewed through any standard web browser by appending the LSID to the prefix http://zoobank.org/.

### ﻿Scientific field survey permission information

Indonesia’s field surveys were approved under the collaborative project between the School of Life Sciences, Nanchang University (China) and Research Center for Biosystematics and Evolution, National Research and Innovation Agency, (Indonesia), in-situ survey certificate (B-3627/IPH.1.02/KS.01.04/IX/2019), and the Non-Commercial Biological Material Transfer Agreement (No. B-1512/IPH.1/KS.01.04/XII/2020).

## Supplementary Material

XML Treatment for Betta iaspis

XML Treatment for Betta mulyadii

## ﻿Additional information

### Author ORCIDs

Jiali Ding https://orcid.org/0009-0009-1322-1666

Wen Lei https://orcid.org/0009-0005-3180-7784

Haryono Haryono https://orcid.org/0000-0002-5143-6790

Wentian Shi https://orcid.org/0000-0003-0130-7963

Wanchang Zhang https://orcid.org/0000-0003-1040-2516

### Data availability

All of the data that support the findings of this study are available in the main text or Supplementary Information.

## Supplementary materials

Supplementary material 1 Comparative material and additional tables Data type docx Explanation note **table S1.** Detailed information of *Bettacoccina* species and outgroup used in the present study. **table S2**. Genetic distances based on mitochondrial Cyt *b* among different species of *Bettacoccina* complex. https://binary.pensoft.net/file/1330596This dataset is made available under the Open Database License (http://opendatacommons.org/licenses/odbl/1.0/). The Open Database License (ODbL) is a license agreement intended to allow users to freely share, modify, and use this Dataset while maintaining this same freedom for others, provided that the original source and author(s) are credited.Jiali Ding, Wen Lei, Haryono Haryono, Wentian Shi, Wanchang Zhang

Supplementary material 2 Additional images Data type docx Explanation note **fig. S1.** Paratype illustrations of *Bettaiaspis* sp. nov. NCUMB.65334, 30 specimens, 17.7–23.9 mm SL; same data as for holotype; females in two columns on the left, males in two columns on the right. **fig. S2.** Paratype illustrations of *Bettamulyadii* sp. nov. NCUMB.65326, 28 specimens, 22.4–28.4 mm SL; same data as for holotype; females in two columns on the left; males in two columns on the right. https://binary.pensoft.net/file/1330597This dataset is made available under the Open Database License (http://opendatacommons.org/licenses/odbl/1.0/). The Open Database License (ODbL) is a license agreement intended to allow users to freely share, modify, and use this Dataset while maintaining this same freedom for others, provided that the original source and author(s) are credited.Jiali Ding, Wen Lei, Haryono Haryono, Wentian Shi, Wanchang Zhang
