# Supplementary material for: ﻿Phylogenetic analysis of Bettacoccina complex (Teleostei, Osphronemidae) from Peninsular Malaysia and Sumatra Island with descriptions of two new species
Source: Zookeys. 2025 May 15;1238:161–81. doi: 10.3897/zookeys.1238.142857 (PMC12099314; doi:10.3897/zookeys.1238.142857)
Supplement: Supplementary material 1 — Comparative material and additional tables [file zookeys-1238-161_article-142857__-s001.docx]

**Supplementary Materials**

**Supplementary file 1.** Comparative material

*Betta brownorum —* NCUMB.65302, 8 ex. 18.2.4-22.1 mm SL, Mandor, Kalimantan Barat, Borneo, coll. Frediksen C. and P. Timothy, 2022;

*Betta burdigala —* NCUMB.65310, 5 ex. 17.5-20.3 mm SL, Toboali, Bangka, coll. Team Tranovim, 2020;

*Betta coccina —* NCUMB.65152, 8 ex. 21.4-25.3 mm SL, Duri, Riau, Sumatra, coll. Maliki (Team R. P. Sugiarto), 2019; NCUMB.65156, 4 ex. 16.4-19.5 mm SL, Rantau Panjang, Riau, Sumatra, coll. Maliki (Team R. P. Sugiarto), 2020; NCUMB.65160, 4 ex. 18.6-22.5 mm SL, Rantau Panjang, Riau, Sumatra, coll. Maliki (Team R. P. Sugiarto), 2023; NCUMB.65164, 4 ex. 15.8-31.2 mm SL, Danau Rasau, Jambi, Sumatra, coll. Mulyadi Tjoa Hong Tjai, 2023;

*Betta livida —* NCUMB.65180, 7 ex. 18.1-21.7 mm SL, Tanjung Malim, Selangor, Peninsula Malaysia, coll. Jit Sin, 2018;

*Betta miniopinna —* NCUMB.65202, 10 ex. 19.3-26.1 mm SL, Teluk Sebong, Bintan Island, coll. Mulyadi Tjoa Hong Tjai, 2023;

*Betta persephone —* NCUMB.65230, 8 ex. 13.3-21.3 mm SL, Rengat, Riau, Sumatra, coll. Mulyadi Tjoa Hong Tjai, 2023; NCUMB.65234, 4 ex. 16.6-22.3 mm SL, Lesung, Riau, Sumatra, coll. Mulyadi Tjoa Hong Tjai, 2023; NCUMB.65238, 10 ex. 22.3-27.1 mm SL, Pijon, Jambi, Sumatra, coll. Mulyadi Tjoa Hong Tjai, 2023;

*Betta rutilans —* NCUMB.65386, 11 ex. 15.1-19.9 mm SL, Ngarak, Kalimantan Barat, Borneo, coll. P. Timothy, 2021;

*Betta tussyae —* NCUMB.65188, 4 ex. 20.1-26.3 mm SL, Kuantan, Pahang, Peninsula Malaysia, coll. Jit Sin, 2019.

**Supplementary Table 1.** Detailed information of *Betta coccina* species and outgroup used in the present study

| **Species** | **Accession**  **number** | **Sample site** | **Origin** |
| --- | --- | --- | --- |
| *B. persephone* Jambi | OQ296582 | Jambi, Malaysia | Donation from Mulyadi Tjoa Hong Tjai, 2022 |
| *B. persephone* Jambi | OQ296583 | Jambi, Malaysia | Donation from Mulyadi Tjoa Hong Tjai, 2022 |
| *B. persephone* Jambi | OQ296584 | Jambi, Malaysia | Donation from Mulyadi Tjoa Hong Tjai, 2022 |
| *B. persephone* Jambi | OQ296585 | Jambi, Malaysia | Donation from Mulyadi Tjoa Hong Tjai, 2022 |
| *B. persephone* Jambi | OQ296586 | Jambi, Malaysia | Donation from Mulyadi Tjoa Hong Tjai, 2022 |
| *B. persephone* Johor | OQ296587 | Johor, Malaysia | Trade materials from Jit Sin, 2021 |
| *B. persephone* Johor | OQ296588 | Johor, Malaysia | Trade materials from Jit Sin, 2021 |
| *B. persephone* Johor | OQ296589 | Johor, Malaysia | Trade materials from Jit Sin, 2021 |
| *B. persephone* Johor | OQ296590 | Johor, Malaysia | Trade materials from Jit Sin, 2021 |
| *B. persephone* Johor | OQ296591 | Johor, Malaysia | Trade materials from Jit Sin, 2021 |
| *B. persephone* Malaysia | OQ296592 | Johor, Malaysia | Trade materials from Hermanus, 2018 |
| *B. persephone* Malaysia | OQ296593 | Johor, Malaysia | Trade materials from Hermanus, 2018 |
| *B. miniopinna* Bintan | OQ296579 | Bintan Island | Trade materials from Oktavianus, 2020 |
| *B. miniopinna* Bintan | OQ296580 | Bintan Island | Trade materials from Oktavianus, 2020 |
| *B. miniopinna* Bintan | OQ296581 | Bintan Island | Trade materials from Oktavianus, 2020 |
| *B. miniopinna* Bintan | OQ296594 | Bintan Island | Trade materials from Oktavianus, 2020 |
| *B. miniopinna* Bintan | OR757056 | Type locality, Bintan Island | Donation from Mulyadi Tjoa Hong Tjai, 2023 |
| *B. miniopinna* Bintan | OR757057 | Type locality, Bintan Island | Donation from Mulyadi Tjoa Hong Tjai, 2023 |
| *B. miniopinna* Bintan | OR757058 | Type locality, Bintan Island | Donation from Mulyadi Tjoa Hong Tjai, 2023 |
| *B. miniopinna* Bintan | OR757059 | Type locality, Bintan Island | Donation from Mulyadi Tjoa Hong Tjai, 2023 |
| *B. burdigala* | OQ296569 | Type locality, Bangka Island | Team Tranovim, 2022 |
| *B. burdigala* | OQ296570 | Type locality, Bangka Island | Team Tranovim, 2022 |
| *B. coccina* Sumatra | OQ296571 | Riau, Sumatra Island | Donation from R. P. Sugiarto, 2021 |
| *B. coccina* Sumatra | OQ296572 | Riau, Sumatra Island | Donation from R. P. Sugiarto, 2021 |
| *B. coccina* Malaysia | OQ296573 | Johor, Malaysia | Trade materials from Jit Sin, 2019 |
| *B. coccina* Malaysia | OQ296574 | Johor, Malaysia | Trade materials from Jit Sin, 2019 |
| *B. hendra* | OQ296575 | Palangkaraya, Kalimantan Tengah | Trade materials from Hendry Sutrisno, 2019 |
| *B. hendra* | OQ296576 | Palangkaraya, Kalimantan Tengah | Trade materials from Hendry Sutrisno, 2019 |
| *B. livida* | OQ296577 | Selangor, Malaysia | Trade materials from Eldon Chan, 2023 |
| *B. livida* | OQ296578 | Selangor, Malaysia | Trade materials from Eldon Chan, 2023 |
| *B. persephone* Riau | OQ296595 | Riau, Sumatra Island | Donation from R. P. Sugiarto, 2020 |
| *B. persephone* Riau | OQ296596 | Riau, Sumatra Island | Donation from R. P. Sugiarto, 2020 |
| *B. persephone* Riau | OQ296597 | Riau, Sumatra Island | Donation from R. P. Sugiarto, 2020 |
| *B. persephone* Riau | OQ296598 | Riau, Sumatra Island | Donation from R. P. Sugiarto, 2020 |
| *B. persephone* Riau | OQ296599 | Riau, Sumatra Island | Donation from R. P. Sugiarto, 2020 |
| *B. persephone* Sumatra | OQ296600 | Sumatra Island | Trade materials from Hermanus, 2018 |
| *B. persephone* Sumatra | OQ296601 | Sumatra Island | Trade materials from Hermanus, 2018 |
| *B. rutilans* | OQ296602 | Ambawang, Kalimantan Barat | Donation from Team Frediksen 2018 |
| *B. rutilans* | OQ296603 | Ambawang, Kalimantan Barat | Donation from Team Frediksen 2018 |
| *B. rutilans* | OQ296604 | Ambawang, Kalimantan Barat | Donation from Team Frediksen 2018 |
| *B. rutilans* | OQ296605 | Ambawang, Kalimantan Barat | Donation from Team Frediksen 2018 |
| *B. rutilans* | OQ296606 | Ambawang, Kalimantan Barat | Donation from Team Frediksen 2018 |
| *B. tussyae* | OQ296608 | Pahang, Malaysia | Trade materials from Jit Sin, 2019 |
| *B. tussyae* | OQ296609 | Pahang, Malaysia | Trade materials from Jit Sin, 2019 |
| *Betta iaspis* sp. nov. | OQ269582 | Type locality, Jambi, Sumatra Island | Donation from Mulyadi Tjoa Hong Tjai, 2022 |
| *Betta iaspis* sp. nov. | OQ269583 | Type locality, Jambi, Sumatra Island | Donation from Mulyadi Tjoa Hong Tjai, 2022 |
| *Betta iaspis* sp. nov. | OQ269584 | Type locality, Jambi, Sumatra Island | Donation from Mulyadi Tjoa Hong Tjai, 2022 |
| *Betta iaspis* sp. nov. | OQ269585 | Type locality, Jambi, Sumatra Island | Donation from Mulyadi Tjoa Hong Tjai, 2022 |
| *Betta iaspis* sp. nov. | OQ269586 | Type locality, Jambi, Sumatra Island | Trade materials from Agus, 2022 |
| *Betta iaspis* sp. nov. | OQ269587 | Type locality, Jambi, Sumatra Island | Trade materials from Agus, 2022 |
| *Betta mulyadii* sp. nov. | OQ269577 | Type locality, Riau, Sumatra Island | Donation from Mulyadi Tjoa Hong Tjai, 2022 |
| *Betta mulyadii* sp. nov. | OQ269578 | Type locality, Riau, Sumatra Island | Donation from Mulyadi Tjoa Hong Tjai, 2022 |
| *Betta mulyadii* sp. nov. | OQ269579 | Type locality, Riau, Sumatra Island | Donation from Mulyadi Tjoa Hong Tjai, 2022 |
| *Betta mulyadii* sp. nov. | OQ269580 | Type locality, Riau, Sumatra Island | Donation from Mulyadi Tjoa Hong Tjai, 2022 |
| *Betta mulyadii* sp. nov. | OQ269581 | Type locality, Riau, Sumatra Island | Donation from Mulyadi Tjoa Hong Tjai, 2022 |
| *Betta macrostoma* | KF203836 | Marudi, Malaysia | Trade material from Lum, 2019 |
| *Parosphromenus deissneri* | MZ578943 | Bangka Island | Trade materials from Hendry Sutrisno, 2021 |

**Supplementary Table 2**. Genetic distances based on mitochondrial Cyt *b* among different species of *Betta coccina* complex.

| Species | 1 | 2 | 3 | 4 | 5 | 6 | 7 | 8 | 9 | 10 | 11 | 12 | 13 | 14 | 15 | 16 |
| --- | --- | --- | --- | --- | --- | --- | --- | --- | --- | --- | --- | --- | --- | --- | --- | --- |
| 1_*P. deissneri* |  |  |  |  |  |  |  |  |  |  |  |  |  |  |  |  |
| 2_*B. macrostoma* | 0.2818 |  |  |  |  |  |  |  |  |  |  |  |  |  |  |  |
| 3_*B. burdigala* | 0.2673 | 0.2448 |  |  |  |  |  |  |  |  |  |  |  |  |  |  |
| 4_*B. coccina Malaysia* | 0.2785 | 0.2627 | 0.1433 |  |  |  |  |  |  |  |  |  |  |  |  |  |
| 5_*B. coccina Sumatra* | 0.2787 | 0.2628 | 0.1433 | 0.0015 |  |  |  |  |  |  |  |  |  |  |  |  |
| 6_*B. hendra* | 0.2679 | 0.2553 | 0.1195 | 0.1400 | 0.1406 |  |  |  |  |  |  |  |  |  |  |  |
| 7_*B. iaspis* sp. nov. | 0.2969 | 0.2449 | 0.2082 | 0.2178 | 0.2185 | 0.1923 |  |  |  |  |  |  |  |  |  |  |
| 8_*B. mulyadii* sp. nov*.* | 0.3146 | 0.2549 | 0.2077 | 0.2162 | 0.2169 | 0.2005 | 0.0435 |  |  |  |  |  |  |  |  |  |
| 9_*B. livida* | 0.2765 | 0.2605 | 0401 | 0.0540 | 0.0541 | 0.1335 | 0.2105 | 0.2105 |  |  |  |  |  |  |  |  |
| 10_*B. miniopinna Bintan* | 0.3135 | 0.2652 | 0.2030 | 0.2179 | 0.2186 | 0.1845 | 0.0605 | 0.0675 | 0.2136 |  |  |  |  |  |  |  |
| 11_*B. persephone Jambi* | 0.3130 | 0.2657 | 0.2004 | 0.2148 | 0.2155 | 0.1857 | 0.0632 | 0.0719 | 0.2137 | 0.0176 |  |  |  |  |  |  |
| 12_*B. persephone Johor* | 0.3144 | 0.2629 | 0.2032 | 0.2149 | 0.2156 | 0.1861 | 0.0608 | 0.0669 | 0.2117 | 0.0109 | 0.0196 |  |  |  |  |  |
| 13_*B. persephone Malaysia commercial* | 0.3122 | 0.2647 | 0.2023 | 0.2133 | 0.2140 | 0.1829 | 0.0584 | 0.0653 | 0.2099 | 0.0070 | 0.0169 | 0.0073 |  |  |  |  |
| 14*_B. persephone Riau* | 0.3077 | 0.2507 | 0.2007 | 0.2128 | 0.2135 | 0.1876 | 0.0551 | 0.0589 | 0.2108 | 0.0163 | 0.0237 | 0.0161 | 0.0164 |  |  |  |
| 15_*B. persephone Sumatra commercial* | 0.3086 | 0.2489 | 0.2016 | 0.2126 | 0.2133 | 0.1895 | 0.0540 | 0.0575 | 0.2115 | 0.0194 | 0.0252 | 0.0225 | 0.0192 | 0.0040 |  |  |
| *16_B. rutilans* | 0.2907 | 0.2619 | 0.1137 | 0.1422 | 0.1423 | 0.1062 | 0.1972 | 0.1917 | 0.1430 | 0.1946 | 0.1992 | 0.1963 | 0.1941 | 0.1899 | 0.1903 |  |
| *17_B. tussyae* | 0.2972 | 0.2604 | 0.1420 | 0.1664 | 0.1668 | 0.1551 | 0.2200 | 0.2200 | 0.1695 | 0.2188 | 0.2196 | 0.2206 | 0.2204 | 0.2128 | 0.2116 | 0.1415 |
