# Supplementary material for: ﻿Phylogenetic analysis of Bettacoccina complex (Teleostei, Osphronemidae) from Peninsular Malaysia and Sumatra Island with descriptions of two new species
Source: Zookeys. 2025 May 15;1238:161–81. doi: 10.3897/zookeys.1238.142857 (PMC12099314; doi:10.3897/zookeys.1238.142857)
Supplement: Supplementary material 2 — Additional images [file zookeys-1238-161_article-142857__-s002.docx]

**
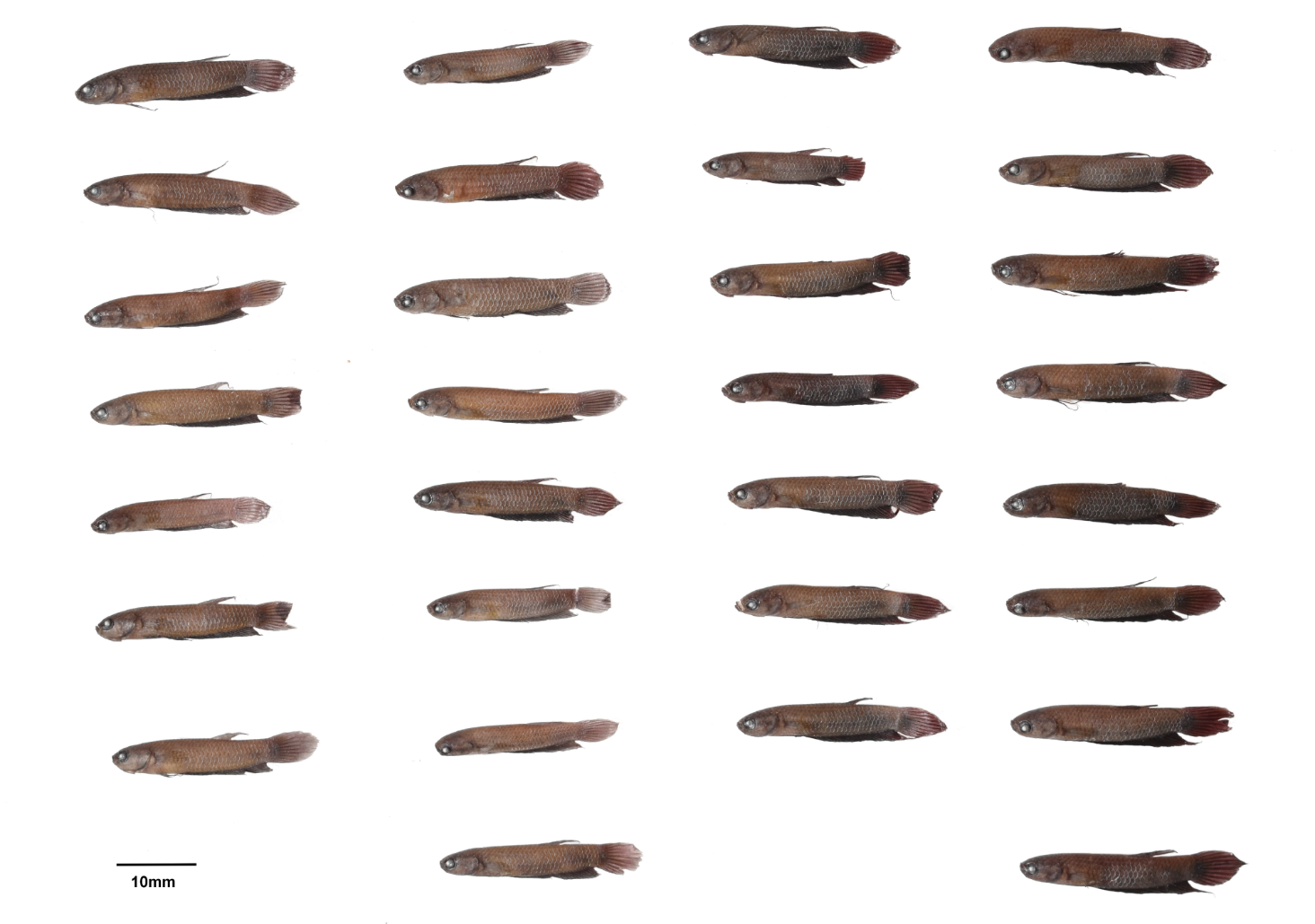
**

**Supplementary Figure 1.** Paratype illustrations of *Betta iaspis* sp. nov. NCUMB.65334, 30 specimens, 17.7-23.9 mm SL; same data as for holotype; females in two columns on the left, males in two columns on the right.

**
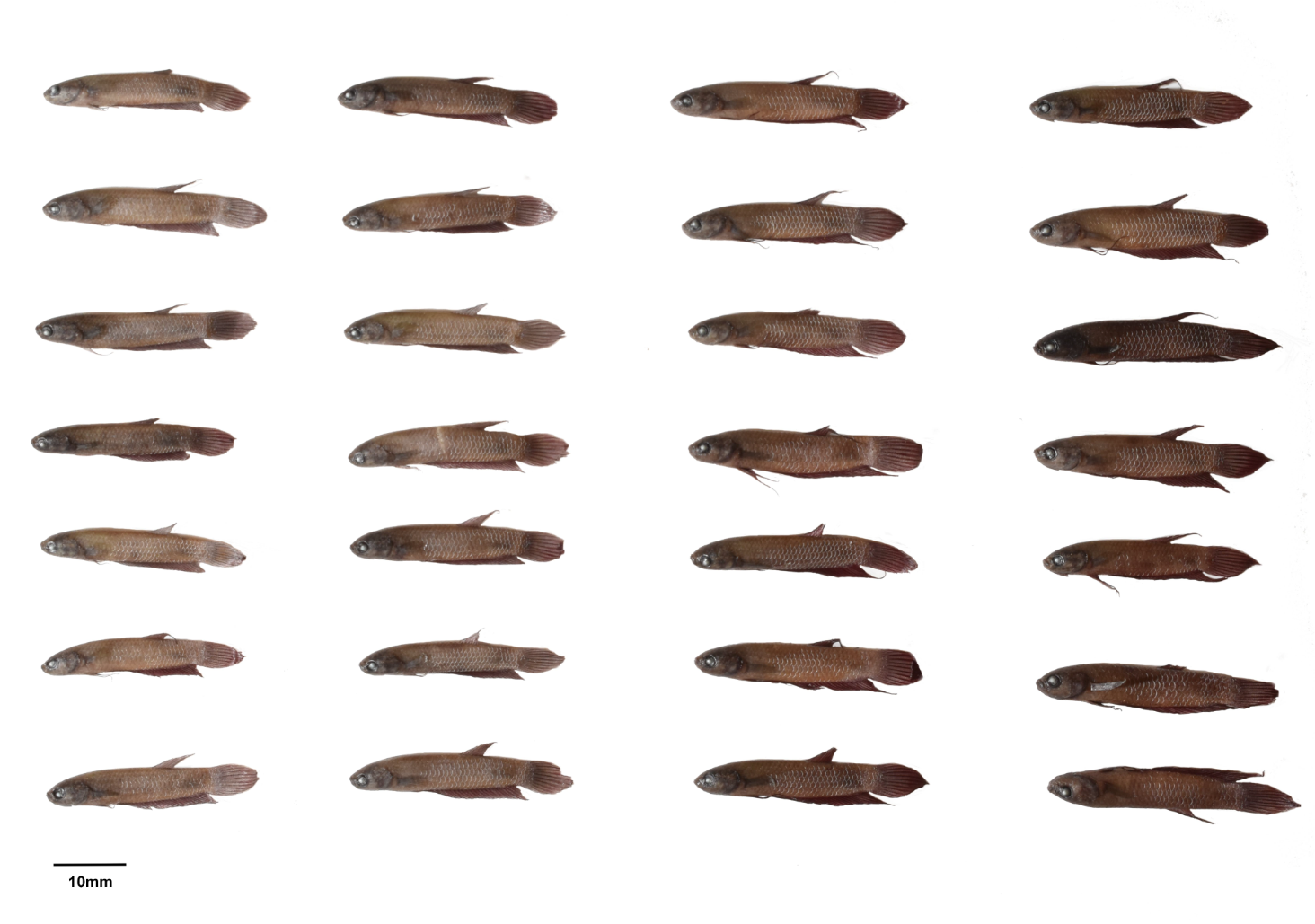
**

**Supplementary Figure 2.** Paratype illustrations of *Betta mulyadii* sp. nov. NCUMB.65326, 28 specimens, 22.4–28.4 mm SL; same data as for holotype; females in two columns on the left; males in two columns on the right.
